# 2-[4-(2,6-Dimeth­oxy­phen­yl)but­yl]-1,3-dimeth­oxy­benzene

**DOI:** 10.1107/S1600536810025420

**Published:** 2010-07-07

**Authors:** Christopher M. Kane, Stephen D. Drake, K. Travis Holman

**Affiliations:** a Department of Chemistry, Georgetown University, 37th and O St NW, Washington, DC 20057, USA

## Abstract

The title compound, C_20_H_26_O_4_, crystallizes such that the alkyl chain adopts an all-*anti* conformation. The crystal packing displays edge-to-face arene–arene inter­actions with a dihedral angle of 87°. The complete molecule is generated by inversion symmetry.

## Related literature

For related compounds containing tethered 2,6-dimeth­oxy­benzene fragments, see: Ionkin *et al.* (2003 ▶); Evans *et al.* (1991 ▶); Yoshimura *et al.* (2008 ▶); Shinohara *et al.* (2008 ▶); Ono *et al.* (2008 ▶). For a related structure, see: Fleck *et al.* (2005 ▶). For the synthesis and further studies, see: Lettré *et al.* (1952 ▶); Tanaka *et al.* (1989 ▶). The rather large crystal used for data collection was chosen in order to optimize data intensity. For weakly absorbing materials, *SADABS* is known to be effective at correcting for crystal sizes larger than the beam without introducing systematic errors, see, for example: Görbitz (1999 ▶).
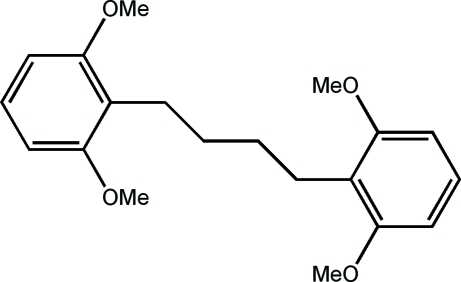


## Experimental

### 

#### Crystal data

C_20_H_26_O_4_
*M* *_r_* = 330.41
Orthorhombic, 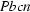

*a* = 22.692 (2) Å
*b* = 5.5460 (5) Å
*c* = 13.7099 (13) Å
*V* = 1725.4 (3) Å^3^
*Z* = 4
Mo *K*α radiation
μ = 0.09 mm^−1^
*T* = 100 K
0.98 × 0.36 × 0.22 mm

#### Data collection

Bruker SMART APEXII CCD diffractometer
Absorption correction: multi-scan (*SADABS*; Bruker, 2001 ▶) *T*_min_ = 0.916, *T*_max_ = 0.981
14196 measured reflections
2071 independent reflections
1853 reflections with *I* > 2σ(*I*)
*R*_int_ = 0.020

#### Refinement

*R*[*F* ^2^ > 2σ(*F* ^2^)] = 0.035
*wR*(*F* ^2^) = 0.098
*S* = 1.05
2071 reflections
161 parameters
All H-atom parameters refined
Δρ_max_ = 0.29 e Å^−3^
Δρ_min_ = −0.17 e Å^−3^

### 

Data collection: *SMART* (Bruker, 2001 ▶); cell refinement: *SAINT* (Bruker, 2001 ▶); data reduction: *SAINT*; program(s) used to solve structure: *SHELXS97* (Sheldrick, 2008 ▶); program(s) used to refine structure: *SHELXL97* (Sheldrick, 2008 ▶); molecular graphics: *X-SEED* (Barbour, 2001 ▶); software used to prepare material for publication: *X-SEED* and *POV-RAY* (Persistence of Vision, 2004 ▶).

## Supplementary Material

Crystal structure: contains datablocks I, global. DOI: 10.1107/S1600536810025420/ng2784sup1.cif

Structure factors: contains datablocks I. DOI: 10.1107/S1600536810025420/ng2784Isup2.hkl

Additional supplementary materials:  crystallographic information; 3D view; checkCIF report

## Figures and Tables

**Fig. 1.**
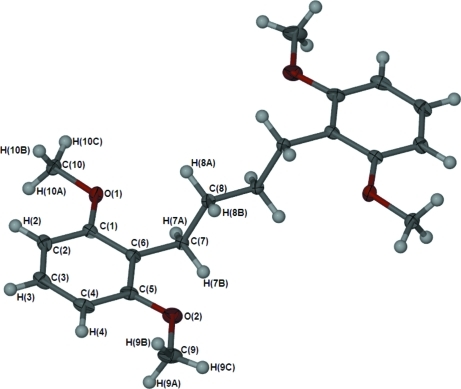
Title compound with atom labels and 50% probability displacement ellipsoids for non-H atoms.

**Fig. 2.**
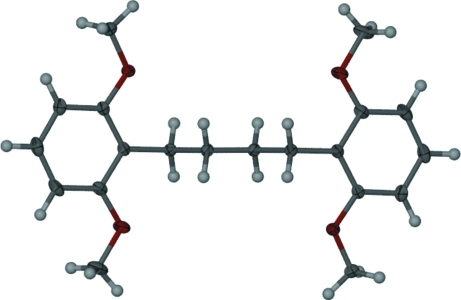
View of title compound along C4 carbon chain.

**Fig. 3.**
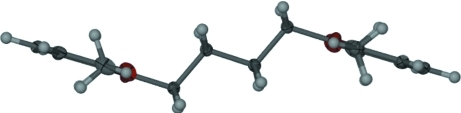
Side view of title compound.

**Fig. 4.**
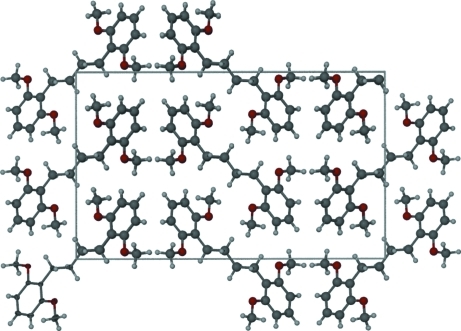
Packing of title compound as viewed down the b axis.

## supplementary crystallographic information

### Comment

Several tethered 2,6-dimethoxyphenyl derivatives have been synthesized containing conjugated linkers comprised of alkenyl and alkynyl units (Yoshimura *et al.*, 2008, Shinohara *et al.*, 2008, and Ono *et. al.*, 2008).

The conformation of the title compound is similar to the hydrocarbon 1,4-diphenylbutane (Fleck *et al.*, 2005). Both molecules exhibit an all *anti* aliphatic conformation. The title compound maintains aromatic C—C bond distances in the range of 1.3844 (16)–1.4042 (13) Å, and aliphatic C—C bonds from 1.5096 (12)–1.5349 (12) Å. One striking difference between the compounds is the crystal packing, which adopts a herringbone pattern for the title compound, whereas in 1,4-diphenylbutane, neither edge-to-face nor π-π stacking interactions are observed.

The rather large crystal (~1 mm) used for data collection was chosen in order to optimize data intensity. For weakly absorbing materials, *SADABS* is known to be effective at correcting for crystal sizes larger than the beam, without introducing systematic errors. See, for example: Görbitz (1999).

### Experimental

The title compound was obtained by lithiation (10 ml, 2.5 M *n*-BuLi in hexanes) of 1,3-dimethoxybenzene (3.45 g, 25 mmol) under nitrogen atmosphere. Following distillation of hexanes and subsequent addition of 1,4-dibromohexane (2.16 g, 10 mmol), the mixture was heated to 150°C for 2 days. After cooling, the mixture was quenched with water (150 ml) and the product was removed and was recrystallized with a 3:1 hexanes/ethyl acetate solution to afford an off-white compound in 72% yield. Single crystals were obtained by slow evaporation from ethanol.

### Crystal data

**Table:**

C_20_H_26_O_4_	*D*_x_ = 1.272 Mg m^−^^3^
*M**_r_* = 330.41	Melting point = 429–431 K
Orthorhombic, *P**b**c**n*	Mo *K*α radiation, λ = 0.71073 Å
Hall symbol: -P 2yn	Cell parameters from 6702 reflections
*a* = 22.692 (2) Å	θ = 3.0–28.5°
*b* = 5.5460 (5) Å	µ = 0.09 mm^−^^1^
*c* = 13.7099 (13) Å	*T* = 100 K
*V* = 1725.4 (3) Å^3^	Prism, colorless
*Z* = 4	0.98 × 0.36 × 0.22 mm
*F*(000) = 712	

### Data collection

**Table:**

Bruker SMART APEXII CCD diffractometer	2071 independent reflections
Radiation source: fine-focus sealed tube	1853 reflections with *I* > 2σ(*I*)
graphite	*R*_int_ = 0.020
φ and ω scans	θ_max_ = 28.0°, θ_min_ = 1.8°
Absorption correction: multi-scan (*SADABS*; Bruker, 2001)	*h* = −29→29
*T*_min_ = 0.916, *T*_max_ = 0.981	*k* = −7→7
14196 measured reflections	*l* = −17→17

### Refinement

**Table:**

Refinement on *F*^2^	Primary atom site location: structure-invariant direct methods
Least-squares matrix: full	Secondary atom site location: difference Fourier map
*R*[*F*^2^ > 2σ(*F*^2^)] = 0.035	Hydrogen site location: inferred from neighbouring sites
*wR*(*F*^2^) = 0.098	All H-atom parameters refined
*S* = 1.05	*w* = 1/[σ^2^(*F*_o_^2^) + (0.0534*P*)^2^ + 0.4726*P*] where *P* = (*F*_o_^2^ + 2*F*_c_^2^)/3
2071 reflections	(Δ/σ)_max_ < 0.001
161 parameters	Δρ_max_ = 0.29 e Å^−^^3^
0 restraints	Δρ_min_ = −0.17 e Å^−^^3^

### Special details

**Table:**

Geometry. All e.s.d.'s (except the e.s.d. in the dihedral angle between two l.s. planes) are estimated using the full covariance matrix. The cell e.s.d.'s are taken into account individually in the estimation of e.s.d.'s in distances, angles and torsion angles; correlations between e.s.d.'s in cell parameters are only used when they are defined by crystal symmetry. An approximate (isotropic) treatment of cell e.s.d.'s is used for estimating e.s.d.'s involving l.s. planes.
Refinement. Refinement of *F*^2^ against ALL reflections. The weighted *R*-factor *wR* and goodness of fit *S* are based on *F*^2^, conventional *R*-factors *R* are based on *F*, with *F* set to zero for negative *F*^2^. The threshold expression of *F*^2^ > σ(*F*^2^) is used only for calculating *R*-factors(gt) *etc*. and is not relevant to the choice of reflections for refinement. *R*-factors based on *F*^2^ are statistically about twice as large as those based on *F*, and *R*- factors based on ALL data will be even larger.

### Fractional atomic coordinates and isotropic or equivalent isotropic displacement parameters (Å^2^)

**Table:**

	*x*	*y*	*z*	*U*_iso_*/*U*_eq_	
C1	0.15557 (4)	0.35467 (17)	0.12621 (6)	0.0182 (2)	
C2	0.19265 (4)	0.29025 (19)	0.20325 (7)	0.0225 (2)	
C3	0.18839 (5)	0.4180 (2)	0.28983 (7)	0.0260 (2)	
C4	0.14842 (5)	0.6043 (2)	0.30147 (7)	0.0257 (2)	
C5	0.11173 (4)	0.66605 (18)	0.22332 (7)	0.0216 (2)	
C6	0.11502 (4)	0.54382 (17)	0.13379 (6)	0.0182 (2)	
C7	0.07619 (4)	0.61358 (17)	0.04894 (7)	0.0185 (2)	
C8	0.01905 (4)	0.46605 (17)	0.04386 (7)	0.0190 (2)	
C9	0.06764 (6)	0.9829 (2)	0.31571 (9)	0.0354 (3)	
C10	0.19556 (4)	0.04010 (18)	0.02716 (8)	0.0229 (2)	
H2	0.2201 (6)	0.161 (2)	0.1972 (9)	0.028 (3)*	
H3	0.2143 (5)	0.375 (2)	0.3442 (10)	0.030 (3)*	
H4	0.1459 (6)	0.689 (3)	0.3613 (10)	0.032 (3)*	
H7A	0.0984 (5)	0.588 (2)	−0.0112 (9)	0.021 (3)*	
H7B	0.0660 (5)	0.785 (2)	0.0535 (8)	0.021 (3)*	
H8A	0.0292 (5)	0.292 (2)	0.0404 (8)	0.021 (3)*	
H8B	−0.0035 (5)	0.491 (2)	0.1046 (8)	0.021 (3)*	
H9A	0.1055 (6)	1.065 (2)	0.3276 (10)	0.031 (3)*	
H9B	0.0560 (7)	0.878 (3)	0.3713 (12)	0.051 (4)*	
H9C	0.0387 (7)	1.102 (3)	0.3050 (11)	0.043 (4)*	
H10A	0.2369 (6)	0.091 (2)	0.0350 (8)	0.025 (3)*	
H10B	0.1868 (6)	−0.085 (2)	0.0749 (10)	0.030 (3)*	
H10C	0.1886 (6)	−0.025 (3)	−0.0395 (10)	0.035 (4)*	
O1	0.15679 (3)	0.24072 (13)	0.03720 (5)	0.02170 (18)	
O2	0.07098 (3)	0.84683 (14)	0.22717 (5)	0.0297 (2)	

### Atomic displacement parameters (Å^2^)

**Table:**

	*U*^11^	*U*^22^	*U*^33^	*U*^12^	*U*^13^	*U*^23^
C1	0.0188 (4)	0.0210 (4)	0.0148 (4)	−0.0041 (3)	0.0011 (3)	0.0013 (3)
C2	0.0208 (5)	0.0261 (5)	0.0207 (5)	−0.0034 (4)	−0.0019 (3)	0.0060 (4)
C3	0.0280 (5)	0.0328 (5)	0.0171 (4)	−0.0109 (4)	−0.0043 (4)	0.0065 (4)
C4	0.0314 (5)	0.0309 (5)	0.0148 (4)	−0.0139 (4)	0.0026 (4)	−0.0024 (4)
C5	0.0209 (5)	0.0234 (5)	0.0205 (5)	−0.0075 (3)	0.0050 (3)	−0.0033 (4)
C6	0.0170 (4)	0.0210 (4)	0.0166 (4)	−0.0043 (3)	0.0009 (3)	0.0001 (3)
C7	0.0181 (4)	0.0181 (4)	0.0192 (4)	0.0000 (3)	−0.0007 (3)	−0.0007 (3)
C8	0.0179 (4)	0.0186 (4)	0.0207 (5)	0.0004 (3)	−0.0006 (3)	−0.0004 (3)
C9	0.0369 (6)	0.0351 (6)	0.0341 (6)	−0.0084 (5)	0.0121 (5)	−0.0179 (5)
C10	0.0225 (5)	0.0207 (5)	0.0254 (5)	0.0030 (4)	0.0008 (4)	0.0000 (4)
O1	0.0247 (4)	0.0241 (4)	0.0163 (3)	0.0066 (3)	−0.0014 (2)	−0.0015 (2)
O2	0.0281 (4)	0.0317 (4)	0.0293 (4)	0.0002 (3)	0.0044 (3)	−0.0139 (3)

### Geometric parameters (Å, °)

**Table:**

O1—C1	1.3746 (11)	C7—H7B	0.979 (12)
O1—C10	1.4251 (11)	C7—H7A	0.976 (12)
O2—C5	1.3650 (13)	C10—H10A	0.986 (13)
O2—C9	1.4314 (12)	C10—H10C	0.995 (14)
C5—C4	1.3992 (14)	C10—H10B	0.975 (14)
C5—C6	1.4042 (13)	C4—C3	1.3844 (16)
C8—C8^i^	1.5283 (18)	C4—H4	0.947 (14)
C8—C7	1.5349 (12)	C2—C3	1.3857 (14)
C8—H8A	0.992 (12)	C2—H2	0.955 (13)
C8—H8B	0.987 (11)	C3—H3	0.979 (13)
C6—C1	1.3993 (13)	C9—H9A	0.985 (13)
C6—C7	1.5096 (12)	C9—H9B	0.994 (16)
C1—C2	1.3967 (13)	C9—H9C	0.943 (16)
			
C1—O1—C10	117.20 (7)	H7B—C7—H7A	108.5 (10)
C5—O2—C9	117.13 (9)	O1—C10—H10A	110.7 (7)
O2—C5—C4	123.58 (9)	O1—C10—H10C	105.8 (8)
O2—C5—C6	115.11 (8)	H10A—C10—H10C	110.8 (10)
C4—C5—C6	121.31 (9)	O1—C10—H10B	111.4 (8)
C8^i^—C8—C7	112.48 (9)	H10A—C10—H10B	109.1 (10)
C8^i^—C8—H8A	109.4 (7)	H10C—C10—H10B	109.0 (11)
C7—C8—H8A	109.0 (7)	C3—C4—C5	118.96 (9)
C8^i^—C8—H8B	109.6 (7)	C3—C4—H4	120.7 (8)
C7—C8—H8B	108.9 (7)	C5—C4—H4	120.4 (8)
H8A—C8—H8B	107.3 (10)	C3—C2—C1	118.36 (9)
C1—C6—C5	117.49 (8)	C3—C2—H2	120.4 (7)
C1—C6—C7	121.24 (8)	C1—C2—H2	121.3 (7)
C5—C6—C7	121.26 (8)	C4—C3—C2	121.73 (9)
O1—C1—C2	122.79 (9)	C4—C3—H3	119.2 (8)
O1—C1—C6	115.08 (8)	C2—C3—H3	119.1 (8)
C2—C1—C6	122.12 (9)	O2—C9—H9A	109.7 (8)
C6—C7—C8	113.04 (7)	O2—C9—H9B	110.9 (9)
C6—C7—H7B	109.7 (7)	H9A—C9—H9B	111.9 (12)
C8—C7—H7B	108.7 (7)	O2—C9—H9C	105.9 (9)
C6—C7—H7A	108.2 (7)	H9A—C9—H9C	108.2 (12)
C8—C7—H7A	108.6 (7)	H9B—C9—H9C	110.1 (12)

Symmetry codes: (i) −*x*, −*y*+1, −*z*.
